# DNA methylation and gene expression analysis in adipose tissue to identify new loci associated with T2D development in obesity

**DOI:** 10.1038/s41387-022-00228-w

**Published:** 2022-12-19

**Authors:** Paulina Baca, Francisco Barajas-Olmos, Elaheh Mirzaeicheshmeh, Carlos Zerrweck, Lizbeth Guilbert, Ernesto Carlos Sánchez, Marlen Flores-Huacuja, Rafael Villafán, Angélica Martínez-Hernández, Humberto García-Ortiz, Cecilia Contreras-Cubas, Federico Centeno-Cruz, Lorena Orozco

**Affiliations:** 1 Immunogenomics and Metabolic Disease Laboratory, Instituto Nacional de Medicina Genómica, SS, Mexico City, Mexico; 2 Facultad de Medicina, Alta especialidad en Cirugía Bariatrica, UNAM, Mexico City, Mexico; 3 Clínica Integral de Obesidad, Hospital General Tláhuac, Secretaría de Salud de la CDMX, Mexico City, Mexico

**Keywords:** Type 2 diabetes, Risk factors, Obesity

## Abstract

**Background:**

Obesity is accompanied by excess adipose fat storage, which may lead to adipose dysfunction, insulin resistance, and type 2 diabetes (T2D). Currently, the tendency to develop T2D in obesity cannot be explained by genetic variation alone—epigenetic mechanisms, such as DNA methylation, might be involved. Here, we aimed to identify changes in DNA methylation and gene expression in visceral adipose tissue (VAT) that might underlie T2D susceptibility in patients with obesity.

**Methods:**

We investigated DNA methylation and gene expression in VAT biopsies from 19 women with obesity, without (OND = 9) or with T2D (OD = 10). Differences in genome-scale methylation (differentially methylated CpGs [DMCs], false discovery rate < 0.05; and differentially methylated regions [DMRs], *p* value < 0.05) and gene expression (DEGs, *p* value <0.05) between groups were assessed. We searched for overlap between altered methylation and expression and the impact of altered DNA methylation on gene expression, using bootstrap Pearson correlation. The relationship of altered DNA methylation to T2D-related traits was also tested.

**Results:**

We identified 11 120 DMCs and 96 DMRs distributed across all chromosomes, with the greatest density of epigenomic alterations at the MHC locus. These alterations were found in newly and previously T2D-related genes. Several of these findings were supported by validation and extended multi-ethnic analyses. Of 252 DEGs in the OD group, 68 genes contained DMCs (*n* = 88), of which 24 demonstrated a significant relationship between gene expression and methylation (*p* values <0.05). Of these, 16, including *ATP11A*, *LPL* and *EHD2* also showed a significant correlation with fasting glucose and HbA1c levels.

**Conclusions:**

Our results revealed novel candidate genes related to T2D pathogenesis in obesity. These genes show perturbations in DNA methylation and expression profiles in patients with obesity and diabetes. Methylation profiles were able to discriminate OND from OD individuals; DNA methylation is thus a potential biomarker.

## Introduction

Obesity is a complex multifactorial disease characterized by an imbalance in energy intake and expenditure that results in adipose tissue expansion [1]. This sustained imbalance compromises the capacity of adipose tissue to store lipids, which leads to ectopic fat accumulation followed by an array of metabolic derangements such as insulin resistance and type 2 diabetes (T2D) [2]. In fact, obesity is a major risk factor for T2D. Furthermore, accumulations of visceral adipose tissue (VAT) contribute more to T2D than accumulations of subcutaneous fat [3]. Although these associations are widely recognized at the population level, individuals differ in their susceptibility to the expected obesity comorbidities. The mechanisms underlying these differences have been difficult to explain on the basis of factors related to lifestyle, environment, and genetic predisposition [4].

An additional mechanism underlying the link between obesity and T2D might involve epigenetic factors, which have been linked to interactions between genetic backgrounds and environmental exposures [5]. Several studies have identified genes with alterations in DNA methylation in patients with obesity. Some of those genes were also associated with T2D or glucose homeostasis [6–8]. Thus, it has been suggested that alterations in DNA methylation might drive T2D development. DNA methylation alterations should correlate with changes in the expression of T2D driver genes. However, the interplay between DNA methylation and gene expression is complex, because different genomic regions can exert a variety of influences on a given gene [9]. Some studies have addressed this issue, but studies that have paired DNA methylation and gene expression analyses in the context of T2D are scarce [10–16]. In this study, we aimed to extend our understanding of the pathogenesis of T2D in obesity by investigating DNA methylation differences between VAT samples from obese individuals with and without T2D. Additionally, we examined correlations between these differences in DNA methylation and changes in gene expression profiles, as well as their relationship to T2D-related traits.

## Subjects and methods

### Subjects and sample collection

Female adults with a body mass index ≥35 kg/m^2^ were recruited prior to bariatric surgery in the Comprehensive Surgery Clinics for Obesity and Metabolic Diseases at Tláhuac General Hospital in Mexico City. To minimize confounding factors, only females were included in the study. We excluded subjects with other known endocrine diseases or dysregulated hypertension. A total of 19 participants with obesity were enrolled in this study, including nine individuals without diabetes (OND group), who were classified as controls, and 10 individuals with obesity and T2D (OD group). All patients in the OD group fulfilled the American Diabetes Association criteria for a T2D diagnosis. We collected data on clinical, biochemical, and anthropometric characteristics at the time of surgery (Supplementary Table S1). From the OD patients, nine were under T2D medication: four were treated with both metformin and insulin, four received metformin treatment only, and one was treated only with insulin. One of the OND patients received metformin treatment. VAT biopsies were acquired during bariatric surgery procedures, then immediately stored in RNAlater (Qiagen, Hilden, Germany) at −70 °C until DNA and RNA extraction. The study was conducted according to the guidelines of the Declaration of Helsinki and approved by the Institutional Ethics and Research Committees of Instituto Nacional de Medicina Genómica (C1_29/2011). A written informed consent was obtained from all subjects before their participation.

### DNA methylation analysis

DNA was extracted from 50 mg of VAT with a QIAamp DNA Mini kit (Qiagen, Valencia, CA, USA). DNA quality and quantity were confirmed by the samples having A_260_/A_280_ and A_260_/A_230_ ratios >1.8, measured with a NanoDrop ND-1000 Spectrophotometer v3.5.2 (NanoDrop Technologies Inc., Wilmington, DE, USA). Integrity was verified by electrophoresis in a 1% agarose gel. Methylation analysis was performed with Infinium Human Methylation EPIC BeadChip Arrays (850 K) according to the manufacturer’s protocol (Illumina, San Diego, CA, USA). Raw data were extracted with GenomeStudio software (V2011.1, Illumina), and all samples passed standard quality controls.

### Gene expression analysis

Total RNA extraction was performed from ~150 mg of VAT with the RNeasy Lipid Tissue Mini Kit (Qiagen, Valencia, CA, USA), according to the manufacturer’s protocol. RNA quality and quantity were assessed using a Bioanalyzer 2100 (Agilent, Santa Clara, CA, USA), with all samples having an acceptable RIN score >8. Global gene expression was analyzed with a Clariom S Human Microarray (Affymetrix, Santa Clara, CA, USA).

### Microarray data analysis

All computational and statistical analyses were performed with R, v4.0 [17]. For DNA methylation analyses, Idat files were processed following the Chip Analysis Methylation Pipeline (ChAMP) package [18]. The raw datasets generated were deposited in the Array Express repository, ID no. E-MTAB-11037. Raw β-methylation scores were calculated as the ratio of the methylated probe intensity to the overall intensity, where overall intensity was the sum of methylated and unmethylated probe intensities. β-methylation values ranged from 0 (unmethylated) to 1 (completely methylated) [19]. Filtering of probes was according to ChAMP default parameters, where probes with <3 beads in at least 5% of samples, non-CpG probes, multi-hit probes, and SNP-related probes (list compiled by Zhou’s Nucleic Acids Research paper in 2016) were removed. In addition, as it was a female cohort, all Y chromosome probes were filtered out, yielding 781 385 probes for subsequent analyses. Data were normalized with the beta-mixture quantile normalization method [20]. To assess for sources of variation in our dataset we applied ChAMP singular value decomposition function (SVD) including as covariates age, batch, and treatment (metformin and/or insulin). After SVD analysis, we implemented ChAMP ComBat method to adjust only for significant covariables (age and microarray batch effects). Probe annotations were based on the HumanMethylationEPIC v1.0 B5 Manifest File. A promoter region was defined when a given CpG was located at TSS1500 (1500–200 bp upstream of the transcriptional start site [TSS]), TSS200 (200 bp upstream of the TSS), in the 5’UTR, or in the first exon, while the gene body encompassed the Body, exon-bound and 3′ UTR [21]. To assess differences in methylation between groups, the mean β value for each CpG site was calculated in each patient group. The difference between groups was defined as the delta β, calculated as the mean OND β value minus the mean OD β value. Differential methylation was identified using Limma [22], and *p* values were adjusted for multiple testing using the Benjamini and Hochberg method [23]. CpG sites with a false discovery rate <0.05 were considered differentially methylated CpGs (DMCs). To identify differentially methylated regions (DMRs) (*p* value area <0.05), we used the Bumphunter algorithm from ChAMP with the default parameters, which creates clusters with a minimum of 7 probes and a maximum separation gap of 300 bp to identify DMRs. Next, to estimate gene expression, CEL files generated from Affymetrix Clariom S human arrays were preprocessed, implementing the oligo Bioconductor package [24]. Raw expression datasets obtained were deposited in the Array Express repository, ID no. E-MTAB-11841. Then, to improve the normalization, we processed them with available blood-sample data [25] obtained from the same array. We applied the robust multi-array average algorithm to adjust the raw intensities. After normalization, we corrected batch effects and age by implementing ComBat. Gene probes encoded in the Y chromosome were removed to obtain expression data for 19,872 probe sets for subsequent analyses. Annotations were obtained from the manufacturer´s website. The differences in gene expression between OND and OD were examined using Limma [22]. Results were listed as log fold change (logFC) with the *p* values adjusted for multiple testing. Benjamini-Hochberg correction yielded no significantly differentially expressed genes (DEGs); therefore, for subsequent analyses, uncorrected *p* values <0.05 were used and, to increase stringency, an additional threshold based on the mean differences between groups with a minimum |logFC|>0.5 was applied.

### Correlation analysis

To evaluate relationships between DMCs or DMRs with their corresponding gene expression (DMC-DEG), as well as their relationship to T2D-related traits (fasting glucose and HbA1c), a Pearson’s correlation was evaluated with a bootstrap analysis, provided in the Boot package, to select only robust associations (*R* = 1000) [26, 27]. In both DMC-DEG and DMC-T2D-related trait correlations, a *p* value <0.05 was considered statistically significant.

### Biological pathway analysis

We analyzed gene overrepresentation with the WEB-based Gene SeT AnaLysis Toolkit (WebGestalt) [28]. Gene symbols that corresponded to DMCs, DMRs, and DEGs were evaluated with the Kyoto Encyclopedia of Genes and Genomes pathways identification analysis. All *p* values were Benjamini-Hochberg adjusted.

### Support of methylation effects

Additional independent DNA methylation data from VAT of 14 Chinese (OND = 8 and OD = 6) and 7 German (OND = 3 and OD = 4) women obtained by EPIC microarray were included to support our methylation findings. We selected all female individuals with data contained in the GSE162166 and E-MTAB-10999 database repositories [13, 14]. Both datasets were combined due to the small size, thus the validation cohort comprised 11 OND and 10 OD subjects. Considering only the CpGs from those DMCs obtained in our initial study, we assessed differential methylation between OND and OD in the combined dataset. To gain statistical power, an additional extended analysis was performed by merging the datasets contained in both public repositories with ours, which yielded a total of 20 OND and 20 OD individuals. Genome-wide DNA methylation profiles were examined following the previously mentioned strategy.

## Results

### Participant characteristics

Clinical characteristics of the participants are detailed in Supplementary Table S1. Body mass index, blood pressure, and lipid serum levels did not differ significantly between the OD and OND groups. As expected, the OD group had significantly (*p* value <0.05) higher HbA1c (6.3 ± 1% vs. 5.4 ± 0.2%) and serum glucose levels (7.49 ± 4.3 mmol/l vs 4.53 ± 0.52 mmol/l) compared with the OND group.

### Differential methylation between OD and OND groups

**Fig. 1:**
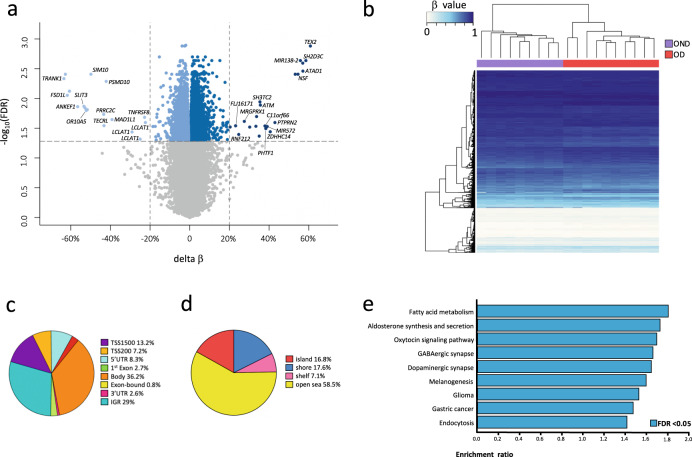
Comparison of visceral adipose tissue DNA methylation profiles between patients with obesity but without diabetes (OND) and patients with obesity and diabetes (OD). **a** Volcano plot shows differences in methylation. Points represent all analyzed CpGs, and blue points indicate DMCs (FDR < 0.05): lighter blue hypomethylated and darker blue hypermethylated in OD, and gray points non-significant. **b** Heat map of DMCs showing DNA methylation levels for each CpG (row) by patient (columns), after applying an unsupervised hierarchical clustering analysis. **c** Distribution of the DMCs among the different genomic locations. **d** Distributions of DMCs across the CGI, the shore (2 kb from the CGI), the shelf (2–4 kb from the CGI), and the open sea (the remaining genome) regions. **e** Gene set enrichment analysis of DMCs (Affinity propagation). Kyoto Encyclopedia of Genes and Genomes pathway enrichments associated with genes with DMCs. *X* axis: FDR (false discovery rate) value. DMCs differentially methylated CpGs, TSS1500 1500–200 bp upstream of the transcriptional start site (TSS), TSS200 200 bp upstream of the TSS, IGR intergenic regions, UTR untranslated region, CGI CpG island.

To identify DNA methylation differences between the OD and OND groups, we compared VAT methylation profiles obtained with Illumina EPIC microarrays. We found DMCs in all chromosomes. The highest and longest density of epigenomic alterations was observed on chromosome 6, with 132 DMCs encompassing 70 genes at the Major Histocompatibility Complex (MHC). Other regions showing high densities of alterations were located on chromosomes 4 and 11 (Supplementary Fig. S1). In the OD group, we found 11 120 DMCs (5880 genes), of which 48.4% were hypomethylated and 51.6% were hypermethylated compared to the OND group (Fig. 1, Supplementary Table S2). An unsupervised hierarchical cluster analysis and multi-dimensional scaling of the DMCs showed a clear methylation profile for each patient group (Fig. 1b, Supplementary Fig. S2).

Notably, DMCs that showed the highest delta β values (>20%) were associated with genes that were newly related to T2D (TRANK1, TEX2, SH2D3C, ATAD1, ANKEF1, MIR138-2, OR10A5, SIM1, PRRC2C, TECRL, ZDHHC14, PHTF1, C11orf66, SH3TC2, MRGPRX1, RNF212, and FLJ16171) or previously related to T2D (FSD1L, NSF, SLIT3, PTPRN2, PSMD10, MAD1L1, MIR572, ATM, LCLAT1, and TNFRSF8) (Supplementary Table S13). Among the 11 120 DMCs, 71% were intragenic, including 39.6% that were mainly distributed in the gene body and 31.4% that were in promoter regions, upstream of the TSS (Fig. 1c). Most DMCs were found in regions with low CpG content, like the shore (17.6%), shelf (7.1%), and open sea regions (58.5%), compared with CpG islands (16.8%; Fig. 1d).

**Fig. 2:**
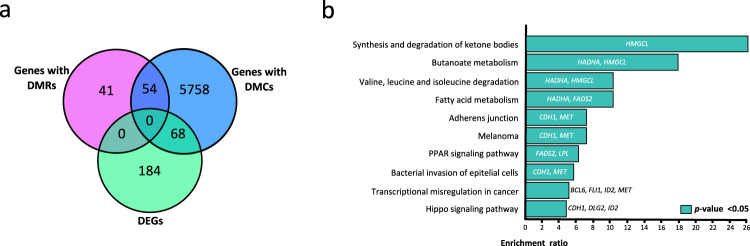
Overlap of differential methylation and gene expression. **a** Venn diagram showing overlap between genes with DMCs or DMRs and the DEGs. **b** Gene set enrichment analysis of overlapping DMC-DEG genes. Kyoto Encyclopedia of Genes and Genomes pathway enrichments associated with genes with DMCs. *X* axis: *p* value. DMCs differentially methylated CpGs, DMRs differentially methylated regions, DEGs differentially expressed genes.

Using the Bumphunter algorithm, we found 96 DMRs that were mainly hypermethylated (74%) in the OD compared to the OND group. Most of these DMRs had overlapping CpG-rich regions (CpG islands; Supplementary Table S3). Furthermore, 92 of the 96 DMRs were located within gene regions. Of these DMRs, 80 were in the vicinity of a TSS, including some that extended into the gene body, and 12 were confined to gene bodies. Additionally, overlaps between DMCs and DMRs were observed in 54 genes, the most significant being in *BLCAP*, *SLC25A24*, *PM20D1*, *PAX8*, and *LCLAT1* (Fig. 2a and Supplementary Table S13).

Among the genes with DMCs, the enrichment analysis mainly identified pathways related to fatty acid metabolism, aldosterone synthesis and secretion, the oxytocin signaling pathway, GABAergic synapse, and dopaminergic synapse, among others (Fig. 1e and Supplementary Table S4). After FDR correction, enrichment analysis of genes with DMRs was not able to identify any significantly enriched pathways.

### Overlapping changes between DNA methylation and gene expression (DMC-DEG)

Gene expression analysis identified 252 DEGs between the OD and OND, with 55.6% being overexpressed and 44.4% underexpressed in the OD (Supplementary Fig. S3 and Supplementary Table S5). Overlap between altered expression and methylation was observed with DMCs, but not with DMRs (Fig. 2a and Supplementary Table S6). Out of the 252 DEGs, 68 (DMCs = 88) showed altered methylation (DMC-DEG); in 35 it was located in the promoter region, and in 53 it was in the gene body. Among those genes with promoter DMCs, 12 were hypomethylated (8 overexpressed and 4 underexpressed) and 23 were hypermethylated (12 overexpressed and 11 underexpressed). In addition, among the genes with DMCs in the gene body, 25 were hypomethylated (8 overexpressed and 17 underexpressed) and 28 hypermethylated (16 overexpressed and 12 underexpressed). Enrichment analysis of the 68 overlapping genes did not reveal any significant pathways at FDR < 0.05. However, with a nominal *p* value (<0.05), we found important enriched pathways such as PPARG and Hippo signaling (Fig. 2b and Supplementary Table S7). Other important pathways were synthesis and degradation of ketone bodies; butonate metabolism; valine, leucine, and isoleucine degradation; and fatty acid metabolism, among others.

### Correlation between methylation and gene expression

**Fig. 3:**
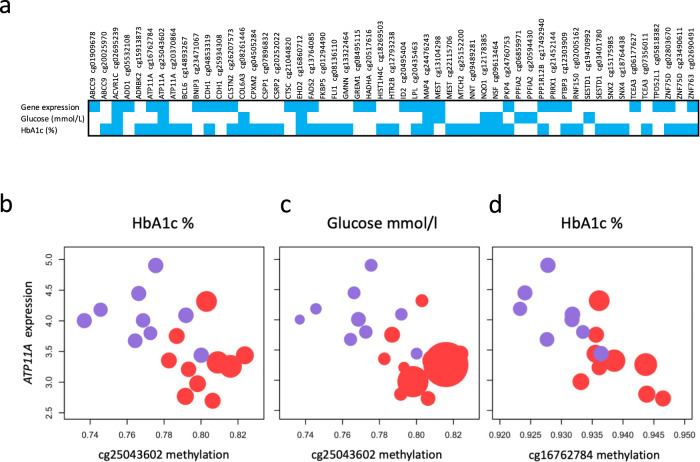
Correlations between differentially methylated CpGs, gene expression, and T2D-related traits. **a** Plot displaying only the DMCs with significant Pearson correlation (*p* value <0.05) between DNA methylation levels and corresponding gene expression, fasting glucose, and HbA1c levels; blue squares indicate those with significant *p* values. **b**–**d** *ATP11A*: Pearson’s correlation for two of its representative DMCs with expression levels. Red dots represent OD patients, purple represents OND; the size of the dot depends on the value of each T2D-related trait. *X* axis: β-methylation; *Y* axis: *ATP11A* expression levels. **b** cg25043602-*ATP11A* expression: *r* = –0.533, *p* = 0.037; cg25043602-HbA1c: *r* = 0.686, *p* = 0.002. **c** cg25043602-Glucose: *r* = 0.539, *p* = 0.031; **d** cg16762784-*ATP11A* expression: *r* = −0.766, *p* = 0.003; cg16762784-HbA1c: *r* = 0.548, *p* = 0.038.

To investigate correlation between expression and methylation, we matched the β-methylation values and expression levels of the DMC-DEG. Among the 88 DMCs (68 genes), we observed 26 (24 genes) with a significant DMC-DEG correlation (Supplementary Table S6). The top five correlations were observed in the genes *ATP11A, LPL*, *PRRX1, ABCC9*, and *EHD2* (Fig. 3 and Supplementary Table S13).

### Identification of correlations between differential methylation and T2D-related traits

To identify the potential relationships of DNA methylation with HbA1c and fasting glucose, we performed Pearson’s correlation analysis using CpG β-methylation values on genes showing DMC-DEG. From 88 DMCs, we found 38 (35 genes) significant correlations with HbA1c (*p* value <0.05), of which 11 were also correlated with fasting glucose (Supplementary Table S6). Notably, from the 24 genes with DMC-DEG correlation, the methylation of 16 genes, including *ATP11A, LPL*, *EHD2, ACVR1C,* and *MAP4*, was also significantly correlated with T2D-related traits (Fig. 3 and Supplementary Table S13).

### Support of methylation analyses: validation and extended analyses

To support the methylation findings, we performed a validation analysis of the DMCs combining two female public datasets (11 OND and 10 OD) [13, 14]. When CpGs from the DMCs obtained in our initial study were contrasted between OND and OD included in the validation dataset, we observed 233 CpGs showing the same effect directions of differential methylation, at a nominal *p* value (<0.05) (Supplementary Table S8). These genes also enriched the glutamatergic synapse, long-term depression, and Hippo signaling pathways (Supplementary Table S9), similar to those observed in our sample.

Additionally, to gain statistical power in our findings, we performed a multi-ethnic extended analysis, combining the datasets contained in both public repositories with our own. When we compared the OD (*n* = 20) and the OND (*n* = 20) groups of the extended cohort, we found 9 648 DMCs in 5 135 genes (Supplementary Fig. S4 and Supplementary Table S10). Out of these, 2 092 genes and 945 DMCs were also found when our cohort was independently analyzed (Supplementary Table S2). All of the shared DMCs showed consistent directionality in both analyses, except for cg25140607 at *TFAP2A* (Supplementary Table S13). Similar to what was observed in our group of Mexican patients, in the multi-ethnic extended cohort the unsupervised hierarchical cluster analysis of the DMCs was able to separate OND and OD patients independently of their ethnic background (Supplementary Fig. S4b). Again, *LCLAT1* displayed multiple DMCs and showed the highest delta β values (>25%), together with *GSTTP2*/*GSTT1* (Supplementary Table S13). Furthermore, the enrichment analysis revealed 26 pathways shared between the two analyses, including oxytocin signaling, GABAergic synapse, glutamatergic synapse, Hippo signaling pathway, MAPK signaling pathway, circadian entrainment, aldosterone synthesis, and secretion, among others (Supplementary Table S11). In addition, 32 DMRs (*GALR1, LCLAT1, SLC25A24, SLC1A2, GRIK2, TDRD12, MIR886, GSTO2, LRCOL1*, etc.) were found in the same genes as the DMRs observed in our cohort (Supplementary Tables S12, S13).

## Discussion

Obesity constitutes a serious health issue because it increases the risk of developing T2D and other comorbidities [29]. A growing body of evidence has shown that perturbations in DNA methylation patterns can contribute to dysfunctional adipose tissue in obesity by inducing changes in gene expression, although the exact mechanisms remain to be understood [8]. A more comprehensive picture of the functional consequences of altered DNA methylation may provide insight into new biological mechanisms underlying tissue dysfunction and lead to improved methods for identifying which individuals with obesity are at risk of progressing to diabetes. In this study, we examined VAT DNA methylation and expression profiles in patients with obesity and compared these profiles between groups with and without T2D. We also documented the correlation of altered methylation with T2D traits.

We found 11 120 DMCs and 96 DMRs between OD and OND individuals. These numbers are higher than those reported in previous studies [11, 13, 14, 30, 31]. Most differential alterations were in regions of low CpG density, like shores and shelves, where methylation has been shown to be relatively dynamic [32, 33]. The DMCs within genes were mainly located in the gene body, and the DMRs were mostly found upstream of the TSS.

Some DMCs and DMRs with the highest delta β values occurred in genes that were previously shown to be altered in T2D. These genes have been involved in insulin resistance and secretion (*ATM, PTPRN2, PSMD10,* and *NSF*), adipogenesis (*SLC25A24* and *PAX8*), inflammatory processes (*TNFRSF8* and *SLIT3*), and mitochondrial processes (*PM20D1* and *LCLAT1*). Others such as *FSD1L* have been associated with T2D in GWAS studies, although the underlying mechanism remains unclear. On the other hand, herein we also report a subset of new genes with altered methylation in OD patients, when compared with OND individuals. Even though their alteration has not been well documented in T2D, some of them have been involved in T2D-related processes such as mitochondrial quality control (*ATAD1*), lipid metabolism (*TEX2* and *TECRL*), inflammation (*TRANK1*) and stress granule assembly (*PRRC2C*), as well as neuronal cells development in the hypothalamus (*SIM1*). In addition, other genes have been involved in cancer processes, such as interference with innate immune system (*SH2D3C*), apoptosis (*BLCAP*), crossing-over regulation during meiosis (*RNF212*), and tumor suppression (*ZDHHC14, MIR138-2,* and *PHTF1*). Finally, another group of genes whose function remains to be further characterized was also found (*ANKEF1, OR10A5, C11orf66*, *SH3TC2*, *MRGPRX1,* and *FLJ16171*) (Supplementary Table S13).

Additionally, the OND and OD groups showed differential DNA methylation along all chromosomes. Although there is little evidence that epigenetic changes in the MHC locus might be involved in T2D [5, 11, 31], in the present study this region showed the greatest and longest density of epigenomic alterations, including in our extended multi-ethnic analysis. Multiple alterations were found in the human leukocyte antigen (HLA) region. Remarkably, similar findings were observed during adipogenesis in vitro from mesenchymal stem cells isolated from the same biopsies analyzed in the present study, suggesting that our observations are derived from the adipocytes and not from the infiltrating inflammatory cells whose numbers are increased in obesity [16]. In addition, a recent study compiling methylation data from adipose tissue found the most altered methylation at HLA in metabolically unhealthy patients with obesity [6]. Together, these findings increase the evidence that the most important genetic region in inflammation and innate immunity, the MHC locus, could be involved in the physiopathology of T2D in patients with obesity, due to epigenetic impairment. As reported in other studies, here the pairing of altered methylation and expression at the MHC locus was difficult to demonstrate. Further exhaustive studies might be required in order to better understand the role of epigenetic regulation in this locus, considering the complexity of its regulatory system due to its high SNV frequency.

Another notable region was the *LCLAT1* locus, which harbors one of the top DMRs and displays multiple DMCs with the highest delta β. Hypomethylation at *LCLAT1* was also found in our validation and multi-ethnic extended study, as well as in other studies, such as the previously mentioned adipogenesis study and that published by Jin-N et al. [13], where the same hypomethylated DMR was documented in T2D patients. Although we did not find any significant relationship between DMC-DEG, there is evidence showing that *LCLAT1* upregulation by oxidative stress and diet-induced obesity in mice reduces insulin-stimulated Akt phosphorylation, leading to insulin resistance and hyperglycemia [34]. This might shed light on the role this gene plays in T2D development.

It is noteworthy that some of the loci with the largest changes in methylation, like the MHC region and *LCLAT1*, and others such as *BLCAP*, *SLC25A24*, *PM20D1, TRANK1, FSD1L*, and *TEX2*, did not show significant changes in their expression. Multiple studies have shown that only a small percentage of transcriptional regulation is dependent on methylation changes [35, 36]. Our findings support this notion, as only a small subset of the genes with DMCs were DEGs. These DMC-DEG were enriched in pathways involved in T2D development, such as PPARG signaling and multiple metabolism-related pathways that promote adipocyte differentiation and mediate insulin sensitization [37–39].

Furthermore, significant correlations were found between multiple DMC-DEG. Notably, the *ATP11A*, *LPL,* and *EHD2* genes displayed the highest methylation-expression correlation. These genes also showed a significant correlation between altered methylation and T2D-related traits. *ATP11A* belongs to a family of 14 P4 ATPases which actively flip phospholipids across cell membranes [40]. Methylation of this gene has been suggested to be involved in colorectal cancer and Crohn’s disease [41, 42]. Even though *ATP11A*’s involvement in obesity and T2D is unknown, there is evidence that deficient expression of some P4 ATPase family members, such as *ATP10C* and *ATP10A*, affects insulin-stimulated mobilization of GLUT4 vesicles to the plasma membrane or the regulation of insulin signaling [43]. Here, *ATP11A* hypermethylation correlated significantly with decreased expression levels and increased fasting glucose and HbA1c levels, suggesting that, similar to *ATP10*, its decreased expression could be related to impaired glucose metabolism.

On other hand, *LPL* encodes lipoprotein lipase, an insulin-dependent rate-limiting enzyme for the hydrolysis of fatty acids. In a previous study, a DMC in the *LPL* promoter was found in VAT from individuals with metabolic syndrome and was positively associated with a worse metabolic profile [44]. Another study documented the most significant signals of association between *LPL* methylation levels in white blood cells and insulin sensitivity measurements [45]. Here, hypermethylation of the *LPL* promoter correlated significantly with an increased HbA1c. Thus, we have provided more evidence that *LPL* hypermethylation can predispose to metabolic diseases like T2D. Similarly, *EHD2*, the EH domain-containing 2, is a known obesity-associated gene implicated in GLUT4 endocytosis and in the maintenance of intracellular lipid metabolism in adipocytes [46, 47]. Its methylation has only been found as altered in epididymal adipocytes of mice with obesity [48]. In our study, a DMC located at *EHD2* body had a significant correlation with its overexpression, and notably, with fasting glucose and HbA1c levels.

In summary, our findings have increased the repertoire of candidate genes involved in mechanisms underlying T2D pathophysiology in patients with obesity, through the analysis of the methylome and its correlation with the transcriptome of adipose tissue and T2D-related traits. Our results suggest that even though the expected correlation between methylation and expression was not observed in multiple genes, methylation impairment is still important for T2D development. Likewise, our findings also support the idea that DNA methylation is a better biomarker than gene expression, since gene clustering analysis was able to discriminate the OND group from the OD group by methylation profiles, but not by expression profiles. Nevertheless, future studies analyzing male samples might strengthen our findings. Even more, some of these marks (MHC region and *LCLAT1*) were shared across individuals with different ethnic backgrounds, as shown by our validation and extended analysis, although some others such as *ATP11A*, *LPL*, *BLCAP*, etc., were found only in our population. Our findings support the notion that methylation profiles are partially shared between different ethnicities, perhaps due to genetic or environmental differences among populations that significantly contribute to shaping the epigenomic susceptibility to disease [49].

Follow-up functional studies will be needed to characterize the pathogenic influence of these alterations and how they contribute to diabetic phenotypes.

## Supplementary information

Supplementary dataset 1

Supplementary figures 1,2,3 and 4; and supplementary table 13

## Data Availability

DNA methylation and Gene expression datasets generated and analyzed during the current study are available in the Array Express repository, accession numbers: E-MTAB-11037 and E-MTAB-11841, respectively.
