# Supplementary material for: DNA methylation and gene expression analysis in adipose tissue to identify new loci associated with T2D development in obesity
Source: Nutr Diabetes. 2022 Dec 19;12:50. doi: 10.1038/s41387-022-00228-w (PMC9763387; doi:10.1038/s41387-022-00228-w)
Supplement: Supplementary file 2 — Supplementary figures 1,2,3 and 4; and supplementary table 13 [file 41387_2022_228_MOESM2_ESM.docx]

**SUPPLEMENTARY MATERIAL**

**Supplementary figures**


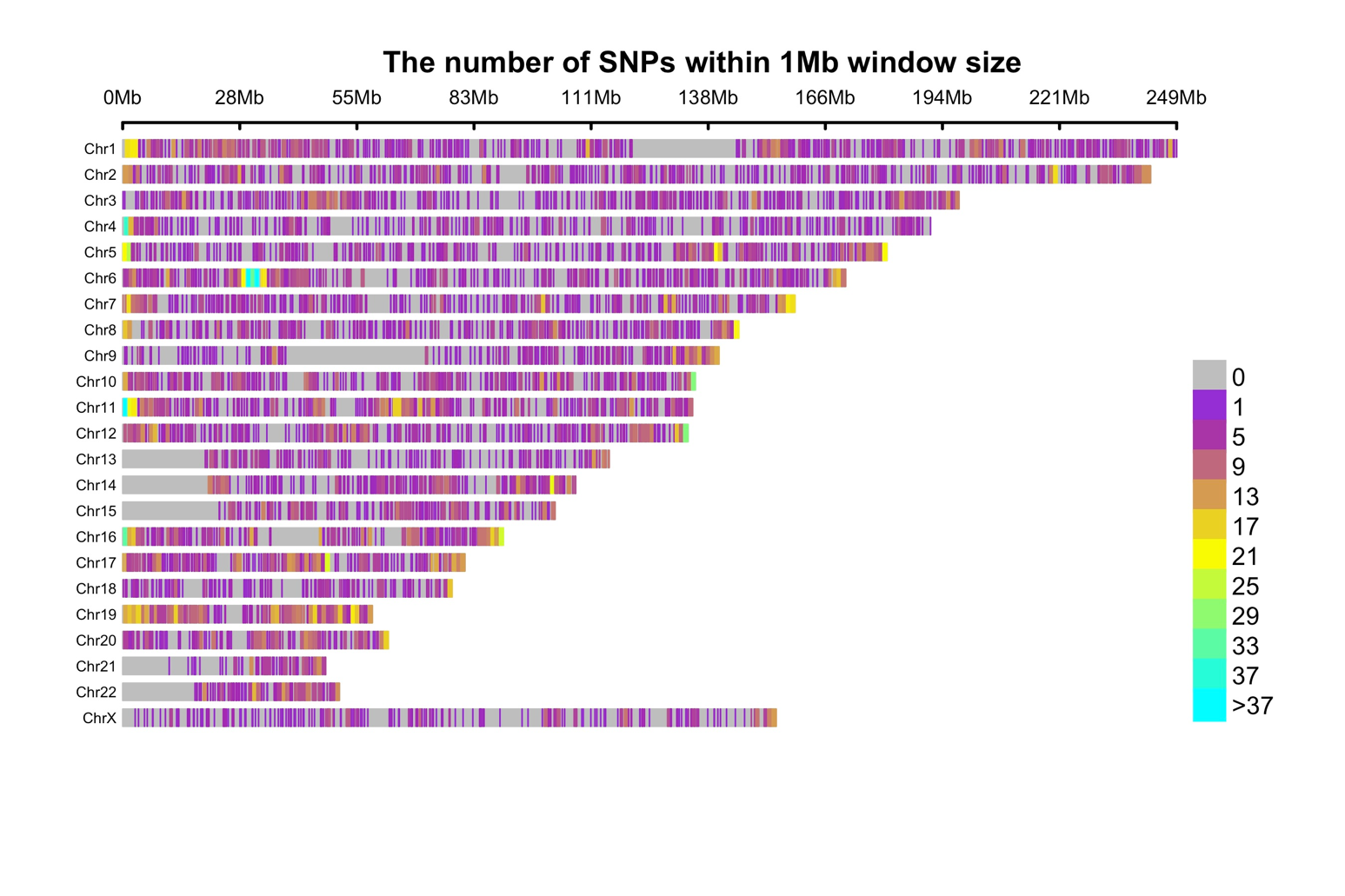


**Supplementary Figure 1. CpG-density plot, according to chromosome, represents the number of DMCs and CpGs within DMRs in a 1-Mb window size.** The horizontal axis shows the chromosome length (Mb); the different colors depict different CpG densities in a 1-Mb window size.

**Supplementary Figure 2. Clustering and multi-dimensional scaling (MDS) of samples, based on differentially methylated CpGs.** MDS of samples, based on methylation at 11 120 differentially methylated CpGs (DMCs). OND, patients with obesity, but without diabetes; OD, patients with obesity and diabetes.

**b**

**a**

**Supplementary Figure 3. Comparison of visceral adipose tissue gene expression profiles between patients with obesity, but without diabetes (OND) and patients with obesity and diabetes (OD). (a)** Volcano plot shows differences in expression. Points represent all analyzed transcripts, green points indicate DEG (logFC >0.5 and *p-*value <0.05), dark green overexpressed and light green underexpressed in OD, and gray non-significant. **(b)** Heat map of DEGs shows expression levels for each gene (row) by patient (columns), after applying an unsupervised hierarchical clustering analysis.

**Supplementary Figure 4. Extended comparison analysis of visceral adipose tissue DNA methylation profiles between patients with obesity, but without diabetes (OND) and patients with obesity and diabetes (OD). (a)** Volcano plot shows differences in methylation. Points represent all analyzed CpGs, orange points indicate DMCs (FDR < 0.05) and black non-significant **(b)** Heat map of DMCs shows methylation levels for each CpG (row) by patients (columns), after applying an unsupervised hierarchical clustering analysis. **(c)** CpG-density plot, according to chromosome, represents the number of DMCs and CpGs within DMRs in a 1-Mb window size. The horizontal axis shows the chromosome length (Mb); the different colors depict different CpG densities in a 1-Mb window size.

**Supplementary Table S13. Function of genes with top hits and its relation to T2D**

**Supplementary reference**

1. Espach Y, Lochner A, Strijdom H, Huisamen B. ATM Protein Kinase Signaling, Type 2 Diabetes and Cardiovascular Disease. Cardiovasc Drugs Ther. 2015;29(1):51–8.

2. Yechoor VK, Patti ME, Ueki K, Laustsen PG, Saccone R, Rauniyar R, et al. Distinct pathways of insulin-regulated versus diabetes-regulated gene expression: An in vivo analysis in MIRKO mice. Proc Natl Acad Sci U S A. 2004;101(47):16525–30.

3. Yu T, Acharya A, Mattheos N, Li S, Ziebolz D, Schmalz G, et al. Molecular mechanisms linking peri-implantitis and type 2 diabetes mellitus revealed by transcriptomic analysis. PeerJ. 2019;2019(6):1–19.

4. Lee S. The association of genetically controlled CpG methylation (cg158269415) of protein tyrosine phosphatase, receptor type N2 (PTPRN2) with childhood obesity. Sci Rep. 2019;9(1):1–7

5. Xu B, O’Donnell M, O’Donnell J, Yu J, Zhang Y, Sartor MA, et al. Adipogenic differentiation of thyroid cancer cells through the pax8-PPARγ fusion protein is regulated by thyroid transcription factor 1 (TTF-1). J Biol Chem. 2016;291(37):19274–86.

6. Skuratovskaia DS, Komar A, Vulf M, Quang HV, Shunkin E, Kirienkova E, et al. Tumor necrosis receptor superfamily interact with fusion and fission of mitochondria of adipose tissue in obese patients without type 2 diabetes. Biomedicines. 2021;9(9).

7. Wang YN, Tang Y, He Z, Ma H, Wang L, Liu Y, et al. Slit3 secreted from M2-like macrophages increases sympathetic activity and thermogenesis in adipose tissue. Nat Metab. 2021;3(11):1536–51.

8. Li J, Romestaing C, Han X, Li Y, Hao X, Wu Y, et al. Cardiolipin remodeling by ALCAT1 links oxidative stress and mitochondrial dysfunction to obesity. Cell Metab [Internet]. 2010;12(2):154–65.

9. Chen Y, Umanah GKE, Dephoure N, Andrabi SA, Gygi SP, Dawson TM, et al. M sp1/ ATAD 1 maintains mitochondrial function by facilitating the degradation of mislocalized tail‐anchored proteins. EMBO J. 2014;33(14):1548–64.

10. Tang H, Jiang L, Stolzenberg-Solomon R, Arslan AA, Beane Freeman LE, Bracci P, Brennan P, Canzian F, Du M, Gallinger S, Giles G, Goodman PJ, Kooperberg C, Le Marchand L, Neale RE, Shu XO, Visvanathan K, White E, Zheng W, Albanes D, Andreotti G, Babic A, B WP. Genome-wide gene-diabetes and gene-obesity interaction scan in 8,255 cases and 11,900 controls from Pancreatic Cancer Cohort Consortium and Pancreatic Cancer Case Control Consortium. Cancer Epidemiol. 2020;176(5):139–48.

11. Andrade S, Morais T, Sandovici I, Seabra AL, Constância M, Monteiro MP. Adipose Tissue Epigenetic Profile in Obesity-Related Dysglycemia - A Systematic Review. Front Endocrinol (Lausanne). 2021;12(June).

12. Yan S, Wang T, Huang S, Di Y, Huang Y, Liu X, et al. Differential expression of microRNAs in plasma of patients with prediabetes and newly diagnosed type 2 diabetes. Acta Diabetol. 2016;53(5):693–702.

13. Liu LK, Choudhary V, Toulmay A, Prinz WA. An inducible ER-Golgi tether facilitates ceramide transport to alleviate lipotoxicity. J Cell Biol. 2017;216(1):131–47.

14. Devalla HD, Gélinas R, Aburawi EH, Beqqali A, Goyette P, Freund C, et al. TECRL, a new life‐threatening inherited arrhythmia gene associated with overlapping clinical features of both LQTS and CPVT. EMBO Mol Med. 2016;8(12):1390–408.

15. Lai J, Jiang J, Zhang P, Xi C, Wu L, Gao X, et al. Impaired blood-brain barrier in the microbiota-gut-brain axis: Potential role of bipolar susceptibility gene TRANK1. J Cell Mol Med. 2021;25(14):6463–9.

16. Youn JY, Dunham WH, Hong SJ, Knight JDR, Bashkurov M, Chen GI, et al. High-Density Proximity Mapping Reveals the Subcellular Organization of mRNA-Associated Granules and Bodies. Mol Cell. 2018;69(3):517-532.e11.

17. Zegers D, Beckers S, Hendrickx R, Van Camp JK, De Craemer V, Verrijken A, et al. Mutation screen of the SIM1 gene in pediatric patients with early-onset obesity. Int J Obes. 2014;38(7):1000–4.

18. Yeh YC, Lawal B, Huang CYF, Hsiao M, Huang TH. Identification of nsp3 (Sh2d3c) as a prognostic biomarker of tumor progression and immune evasion for lung cancer and evaluation of organosulfur compounds from allium sativum l. as therapeutic candidates. Biomedicines. 2021;9(11).

19. Daniel Harris, BA, Lynn McNicoll, MD, Gary Epstein-Lubow, MD, and Kali S. Thomas P, Chirag M Lakhani1, Braden T Tierney1, 2, Arjun K Manrai1, 3, Jian Yang4, 5, Peter M Visscher#4, 5,*, and Chirag J Patel#1 1Department. A SUMO-ubiquitin relay recruits proteasomes to chromosome axes to regulate meiotic recombination. Physiol Behav. 2017;176(1):139–48.

20. Yeste-Velasco M, Mao X, Grose R, Kudahetti SC, Lin D, Marzec J, et al. Identification of ZDHHC14 as a novel human tumour suppressor gene. J Pathol. 2014;232(5):566–77.

21. Sha HH, Wang DD, Chen D, Liu SW, Wang Z, Yan DL, et al. MiR-138: A promising therapeutic target for cancer. Tumor Biol. 2017;39(4).

22. Huang X, Geng S, Weng J, Lu Z, Zeng L, Li M, et al. Analysis of the expression of PHTF1 and related genes in acute lymphoblastic leukemia. Cancer Cell Int. 2015;15(1):1–11.

23. Moreira JMA, Ohlsson G, Gromov P, Simon R, Sauter G, Celis JE, et al. Bladder cancer-associated protein, a potential prognostic biomarker in human bladder cancer. Mol Cell Proteomics. 2010;9(1):161–77.

24. Urano T, Shiraki M, Sasaki N, Ouchi Y, Inoue S. SLC25A24 as a novel susceptibility Gene for low fat mass in humans and mice. J Clin Endocrinol Metab. 2015;100(4):E655–63.

25. Benson KK, Hu W, Weller AH, Bennett AH, Chen ER, Khetarpal SA, et al. Natural human genetic variation determines basal and inducible expression of PM20D1, an obesity-associated gene. Proc Natl Acad Sci U S A. 2019;116(46):23232–42.

26. Chu Y, Zhu C, Wang Q, Liu M, Wan W, Zhou J, et al. Adipose-derived mesenchymal stem cells induced PAX8 promotes ovarian cancer cell growth by stabilizing TAZ protein. J Cell Mol Med. 2021;25(9):4434–43.

27. Wang J, Molday LL, Hii T, Coleman JA, Wen T, Andersen JP, et al. Proteomic Analysis and Functional Characterization of P4-ATPase Phospholipid Flippases from Murine Tissues. Sci Rep [Internet]. 2018;8(1):2–15.

28. Castellano-Castillo D, Moreno-Indias I, Fernandez-Garcia JC, Alcaide-Torres J, Moreno-Santos I, Ocana L, et al. Adipose tissue LPL methylation is associated with triglyceride concentrations in the metabolic syndrome. Clin Chem. 2018;64(1):210–8.

29. Dankel SN, Grytten E, Bjune JI, Nielsen HJ, Dietrich A, Blüher M, et al. COL6A3 expression in adipose tissue cells is associated with levels of the homeobox transcription factor PRRX1. Sci Rep [Internet]. 2020;10(1).

30. Guilherme A, Soriano NA, Bose S, Holik J, Bose A, Pomerleau DP, et al. EHD2 and the Novel EH Domain Binding Protein EHBP1 Couple Endocytosis to the Actin Cytoskeleton. J Biol Chem [Internet]. 2004;279(11):10593–605.

31. Fryklund C, Morén B, Shah S, Grossi M, Degerman E, Matthaeus C, et al. EH Domain-Containing 2 Deficiency Restricts Adipose Tissue Expansion and Impairs Lipolysis in Primary Inguinal Adipocytes. Front Physiol. 2021;12(September).

32. Emdin CA, Khera A V., Aragam K, Haas M, Chaffin M, Klarin D, et al. DNA sequence variation in ACVR1C encoding the activin receptor-like kinase 7 influences body fat distribution and protects against type 2 diabetes. Diabetes. 2019;68(1):226–34.

33. Li L, Zhang Q, Lei X, Huang Y, Hu J. MAP4 as a New Candidate in Cardiovascular Disease. 2020;11(August):1–9.

34. Scott CC, Vossio S, Rougemont J, Gruenberg J. TFAP2 transcription factors are regulators of lipid droplet biogenesis. Elife. 2018;7:1–24.

35. Klusek J, Błońska-Sikora E, Witczak B, Orlewska K, Klusek J, Głuszek S, et al. Glutathione S-transferases gene polymorphism influence on the age of diabetes type 2 onset. BMJ Open Diabetes Res Care. 2020;8(2):1–7.

36. Kim A, Park T. Diet-induced obesity regulates the galanin-mediated signaling cascade in the adipose tissue of mice. Mol Nutr Food Res. 2010;54(9):1361–70.

37. Tsai SF, Chen YW, Kuo YM. High-fat diet reduces the hippocampal content level of lactate which is correlated with the expression of glial glutamate transporters. Neurosci Lett [Internet]. 2018;662(October 2017):142–6.

38. T B, Arianti R, Shaw A, Attila V. FTO Intronic SNP Strongly Influences Human Neck Adipocyte Browning Determined by Tissue and PPARγ Specific Regulation: A Transcriptome Analysis. Cells. 2020;9(987).

39. Kim M, Ki BS, Hong K, Park SP, Ko JJ, Choi Y. Tudor domain containing protein TDRD12 expresses at the acrosome of spermatids in mouse testis. Asian-Australasian J Anim Sci. 2016;29(7):944–51.

40. Lee K, Kunkeaw N, Jeon SH, Lee I, Johnson BH, Kang GY, et al. Precursor miR-886, a novel noncoding RNA repressed in cancer, associates with PKR and modulates its activity. Rna. 2011;17(6):1076–89.

41. Menon D, Innes A, Oakley AJ, Dahlstrom JE, Jensen LM, Brüstle A, et al. GSTO1-1 plays a pro-inflammatory role in models of inflammation, colitis and obesity. Sci Rep. 2017;7(1):1–15.
